# Alternatives to Antibiotics in Animal Agriculture: An Ecoimmunological View

**DOI:** 10.3390/pathogens4010001

**Published:** 2014-12-29

**Authors:** Yongming Sang, Frank Blecha

**Affiliations:** Department of Anatomy and Physiology, College of Veterinary Medicine, Kansas State University, Manhattan, KS 66506, USA; E-Mail: blecha@vet.k-state.edu

**Keywords:** alternatives to antibiotics, antibiosis, antibiotic resistance and tolerance, bacterial resisters and persisters, ecoimmunology

## Abstract

Ecological immunology (or ecoimmunology) is a new discipline in animal health and immunology that extends immunologists’ views into a natural context where animals and humans have co-evolved. Antibiotic resistance and tolerance (ART) in bacteria are manifested in antibiosis-surviving subsets of resisters and persisters. ART has emerged though natural evolutionary consequences enriched by human nosocomial and agricultural practices, in particular, wide use of antibiotics that overwhelms other ecological and immunological interactions. Most previous reviews of antibiotic resistance focus on resisters but overlook persisters, although both are fundamental to bacteria survival through antibiosis. Here, we discuss resisters and persisters together to contrast the distinct ecological responses of persisters during antibiotic stress and propose different regimens to eradicate persisters. Our intention is not only to provide an ecoimmunological interpretation, but also to use an ecoimmunological system to categorize available alternatives and promote the discovery of prospective approaches to relieve ART problems within the general scope of improving animal health. Thus, we will categorize available alternatives to antibiotics and envision applications of ecoimmunological tenets to promote related studies in animal production.

## 1. Ecoimmunology: Animal Immunity and Health in the Natural Context

A majority of immunological studies have been conducted under controlled laboratory conditions in a single animal species. Ecological immunology (or ecoimmunology) stems from immunological studies in wild animals to determine the host’s health in natural settings and considers animal immunity as an integral part of organismal biology [1,2,3]. Hallmark studies responsible for the rise of ecoimmunology include: (1) the immunocompetence handicap hypothesis (ICHH) to explain variation in male sexual selection due to the immunoregulation of sex hormones [4,5,6,7]; (2) interaction among neuroendocrine and immune systems to determine mating behavior, aggression, and host–pathogen interaction [8,9,10,11,12]; (3) immune variance and the trade-off between animal immune function and physiological, morphological, and behavioral traits to maximize reproduction and survival [13,14]; and (4) immune variance as a result of habitat change, yearly seasonality, and different life stages [15,16,17,18]. In general, the evolving concepts of ecoimmunology share much with the disciplines of disease ecology, integrative biology, and evolutionary medicine [3,5,17,18]. Since 2009, studies in this area have burgeoned to redefine ecoimmunology as “an interdisciplinary field that investigates the effects of biotic and abiotic factors on the variation in immunity of free-living species” [19]. Thus, “ecoimmunology is an emerging discipline that seeks to understand the physiological, ecological, and evolutionary causes and consequences of variation in immune system” [20]. Ecoimmunology emphasizes enhancing animal immunity at both intra- and inter-organismal levels through ecological interaction rather than mere pathogen killing [2,3,4,5].

Agricultural animals, through domestication and intensive farming practices, can be placed in a special ecological niche (eco-niche) with respect to their immune/physiological systems and interaction with constrained biotic and abiotic components [21,22]. The immunological state of agricultural animals thus requires applying ecological principles to interpret at both intra- and inter-organismal levels, particularly in analyzing the epizootic threats affecting public health [21,22,23,24], including widespread antibiotic resistance/tolerance (ART) and epidemics of zoonotic diseases [25,26,27,28]. Bacteria generally survive antibiotic stress through two mechanisms: active antagonism via obtaining antibiotic-resistant genes (resisters) and adaptive tolerance via growth/metabolic adjustment (persisters) [25,26,27,28,29,30]. The wide use of antibiotics in animal production and medical practices has perturbed animal–microbe (including human–microbe) interaction, causing a large disturbance in this eco-niche and boosting the selection and prevalence of ART [25,26,27,28].

Applying ecoimmunological principles, we have categorized current and prospective alternatives to antibiotics and propose an integrative approach centered on increasing animal immunological phenotypes to restrain the emergence and transfer of ART threats. We have organized available alternatives to antibiotics and envision prospective means of applying ecoimmunological concepts to promote related studies in animal production. Previous reviews of antibiotic resistance focus mostly on resisters [25,26,27,28,31,32,33,34,35]; however, recent studies have revealed distinct ecological responses of persisters during antibiotic stress and proposed different regimens to eradicate persisters [29,30,36].

An evolutionary view of an animal eco-niche within natural settings promotes understanding of human disturbances of animal eco-immune systems. The wild ancestors of current agricultural animals evolved in their local natural settings for nearly 200 million years before domestication by humans [37,38,39]. This eco-niche containing biotic and abiotic components centered on a wild animal can be stratified into three principal layers. An animal eco-niche, even in the smallest ecosystems, includes ecological components for primary production (*i.e.*, plant food resources), energy flow (*i.e.*, animals, including humans in the food chain), and decomposition (*i.e.*, microbes) processes to recycle nutrients [28,37,38,39]. In an ecoimmunological examination, we posit the animal immune system at the center and treat other animal systems and genomic backgrounds as internal factors that interact and contribute to animal immune responses [10,11,12,13,14,20]. Because of their significant role in immune regulation and ART development within the animal, we also treat animal microbiota as a special “internal” system [40,41,42,43,44]. Immediately outside the animal body is a proximal environment that contains abiotic and biotic components (such as chemical odors, aerosols from body secretions, dirt, parasites, and microbes) that buffer and record animal interactions with distal external factors. The outer layer of this eco-niche illustrates that several factors typically interact with animal systems to affect animal health and growth status [28,37,38,39]. In brief, evolutionary constraints and life history (*i.e.*, time factor) refine the genotypes/epigenome underlying the animal’s biological responses, food resources to produce energy and material flow to support the animal’s growth, and microbial ecology tempers potential infections and microbiota alterations [21,22]. Compared with today’s animals, prehistoric animals had limited interaction with humans aside from occasional engagement in a prey–predator relationship as part of the food chain of the ecosystem [37,38,39]. The succession of this natural ecosystem lasted for several million years before domestication and intensification in animal agriculture [37,38,39], which through subsequent use of antibiotics in animal production, led, in part, to selection and enrichment of ART [25,26,27,28,29,30,31,32,33,34,35,36].

## 2. Ecoimmunological View of Antibiotic Resistance and Tolerance

Ecoimmunology seeks to understand the variation in the animal immune system in an ecological and evolutionary context [19]; hence, an ecological and evolutionary examination about natural emerging and artificial enrichment of ART occurrences will set forth essential background to associate ART prevalence with ecoimmunological principles [2,3,4,5]. The spontaneous occurrence of ART mutants and horizontal/vertical transfer of antibiotic-resistant (AR) genes at the inter- and intra-organismal levels have been extensively reviewed elsewhere [25,26,27,28,29,30,31,32,33,34,35,36]. Before human predominance, the eco-niches of wild animals are believed to have functioned very well given the flourishing wild herds that spread across most virgin lands despite periodic population drops because of dramatic climate change [45,46,47]. The first profound human disturbance of natural animal eco-niches was the domestication of agricultural animals from 9000–1000 BC [37,45]. Domestication fundamentally changed the ecological dependence and living environment to which wild animals were accustomed. Early humans of the pre-agricultural era were primarily nomadic settlers; hence, domesticated animals had to adapt to the environments of new habitats, which not only differed from the animals’ natural lairs but also frequently changed [37,45,46,47]. Recent discoveries of the genetic differences between the genomes of wild and agricultural animal breeds in pigs, horses, and cattle have demonstrated the accumulated adaptions of domestication; however, most environmental adaption is reflected only at epigenetic levels, which remain largely elusive [45,48,49,50,51]. Agricultural domestication profoundly changed inter-organismal interaction in wild animals, particularly their ecological relationship with humans. Previous ecological competitors (or occasional predators) became essential regulators, even dominators, of most aspects of the life of domestic animals [37,46]. These effects include living environments subordinate to human communities, mingling in herds with animals of other species or subtypes from other ecotypes, and food resources derived from humans’ leftovers. Inter-organismal interaction became dense not only on the scale of “macro-species” of animals, but also in the exchange of microorganisms. Recent studies have shown that animal microbiota serve as an essential “internal” organ underlying immune and physiological responses [40,41,42,43,44]. Highly controlled breeding practices (e.g., for higher production performance to benefit humans) combined and accrued a series of genetic changes underlying animal immune response to xenobiotics and microbial infections [40,41,42,43,44,45,46,47,48,49,50,51]. Domestic animals inevitably experienced the extension of human medicines and medical practices during sickness (which evolved into professional veterinary practice) or served as experimental animals for undefined tests and medicines, a practice that led to therapeutic and preventive use of antibiotics in agricultural animals since the mid last century [21,22,35]. Finally, the integration of planting and animal farm systems intensively promoted biotic flux (including genetic materials and bioactive metabolites) within the agricultural ecosystem (such as through crops fertilized with animal waste and the recycling of contaminated water and land resources) [21,22,23]. In summary, the domestic eco-niches of animal growth in the pre-industrial era of agriculture compacted both biotic and abiotic factors and promoted the mutations required for ART bursts given the presence of later antibiotic pressure [25,26,27,28,29,30,31,32,33,34,35].

Although ART has apparently increased only in recent decades, the genetic mechanisms of AR (*i.e.*, AR genes or potential pseudogenes) and stochastic persisters evolved in our ecosystem and were enriched by the agricultural system even before the use of antibiotics [25,26,27,28,29,30,31,32,33,34,35]. The definition of “antibiotics,” as first proposed by Selman Waksman (the discoverer of streptomycin), is simply a description of an application and does not refer to the chemical or major biological nature of the compounds [52]. Current antibiotics include all synthetic or natural organic compounds that inhibit or kill bacteria, but archetype antibiotics (such as penicillin and streptomycin) are purified from secondary microbial metabolites [52,53,54]. Highly purified or synthetic analogs of antibiotics were unavailable prior to the industrial era, but antibiotics or their parental molecules are not novel biological agents. Secretion of secondary metabolites is a self-preserving or competitor-repelling technique ubiquitously used by microbial and plant species [54,55,56,57,58,59,60,61,62,63,64,65,66]. Although plant-originated compounds that are microbiostatic or microbicidal are generally called “antimicrobials” rather than antibiotics [57,59], they have similar biotic effects and should be considered pertinent to ART origin and evolution. In an ecosystem, underlying contradictions among interacting components and opposite sides of an interaction propel evolution [2,3,4,5]. The presence of antibiotic metabolites accompanies ART mechanisms. This has been confirmed by the discoveries of antibiotic-resistant mutants nearly simultaneously with or shortly after the introduction of new antibiotics [25,26,27,28,29]. Nevertheless, in a natural context, the “arms race” with antibiotic metabolites in each ecotope evolved “peacefully” in something like a win-win game for millions of years until met with huge disturbances, including those brought by the processes of agriculture and industrialization [25,26,27,28,29,37,38,39,29,37]. Few studies have been conducted at the ecotope level to show whether the agricultural system (*i.e.*, intensive crop and animal production) has promoted the evolution of ART mechanisms, but historical records of disease outbreaks during the traditional agriculture era serve as indirect proof [22,23,24,25,32]. The traditional agricultural system clearly accumulated interactions between antibiotic metabolites and relevant ART mechanisms within individual ecotopes for thousands of years [67,68,69,70,71,72]. Studies have shown that the production of antibiotic metabolites in Streptomycetes and fungi tend to increase during changes in cultural conditions, the presence of unfavorable carbon sources, the absence of phosphate, and shifts in nitrogen source [52,53,54,55,58]; all of these factors have been enriched by animal agriculture and agronomy [21,22,23].

The discovery and wide use of antibiotics represent a major event to change the animal-proximal microbial landscape. Nontherapeutic use of antibiotic products as growth promoters in animal agriculture is among the factors that exacerbate the ART threat [26,28,32,35,72,73,74]. Recent studies have shown that in contrast to high-dose therapeutic uses in hospitals, constant presence of sub-lethal levels of antibiotics in an eco-niche plays an unexpected role in the evolution of AR [31,72]. Two mechanisms contribute to bacterial survival of antibiotic stresses. One is the emergence and directional selection of resisters, which obtain antibiotic-resistant genes to exclude or detoxify antibiotics [25,27,31], and the other is the emergence and enrichment of persisters, which are cells tolerant to antibiotics as they have entered into a dormant or slow-growing state (without genomic changes) [29,30,36]. While nearly 100 AR genes have been identified in bacteria [25,27,31], the stochastic and stimulated (due to physiological and environmental stresses) rise of persisters also causes much of the bacterial survival during antibiotic therapies [29,30]. Therefore, alternative approaches to reducing persister occurrence or reversing persisters to a sensitive status are equally important for dealing with ART problems [29,30,36].

Since the ban on antibiotics used as growth promoters in some European countries, controlled use of antibiotics in animal production has become a trend worldwide and functional alternatives to antibiotics have been developed to replace nontherapeutic uses of antibiotics in animal production [26,28,32,33,35,73,74]. In line with this, systematic categorization of classical and prospective alternatives to antibiotics by applying ecological principles of ecoimmunology will be fundamental to envisioning novel types of alternatives and new functional aspects of classical alternatives [2,3,4,5].

## 3. Ecoimmunological Classification of Alternatives to Antibiotics

For microorganisms (as well as higher organisms), chemical signaling and response to organic xenobiotics are essential for all biological responses and fundamental to evolution of immunity against non-self at both molecular and organismic levels [75,76]. In other words, antibiosis through metabolite-mediated repulsion, inhibition, or killing is a primitive form of innate immunity and comprises a part of metabolism [61,62,63,64,65,66]. This produces the multifunctional property of antibiotic metabolites in both antibiosis and other eco-physiological action [54,58,61,62,63,66]. In general, secondary metabolites including antibiotics serve: (1) as competitive weapons in general antibiosis against other organismic species; (2) as potential immune and metabolic modulators; (3) as metal transporting agents; (4) as agents signaling symbiosis among organismic species; (5) as sexual hormones and plant growth regulators; and (6) as effectors of cell differentiation [66,77,78,79,80,81,82].

Despite their designation as “wonder medicines”, the primary use of antibiotics is to impede pathogenic bacteria to give the host time to increase immunity [53,61,65]. In animal agriculture, the external bacterial communities are closely connected to internal animal microbiota, which works as an animal “organ” to necessitate animal metabolic, physiological, and immune responses [40,41,42,43,44]. Current antibiotics have no way to target pathogenic bacteria without perturbing microbial ecology [83,84,85]. Ecoimmunology thus calls for organizing the most effective approaches at an ecological scale to boost host immunity and intervene in interactions between the host and microorganisms rather than eliminating pathogens alone [86,87,88,89,90,91,92,93]. This approach may therefore provide a system to manage various measures as alternatives to antibiotics [26,28,32,33,35,73,74]. According to their ecological relationship with the animal immune system, we have classified known and prospective alternatives to antibiotics in the order of environmental abiotic factors, biotic factors (pathogenic and not pathogenic), intra-organismal systems of animals, and typical interaction among these factors signified as host–pathogen interaction. Table 1 lists a classification of current and prospective alternatives to antibiotics with elaboration of some prospects related to ecoimmunological disciplines [2,3,4,5]. Note that approaches intended to eradicate bacterial persistence (persisters) are not included in Table 1 [29,30,36] but are specifically discussed in Section 4.

**Table 1:** Current and prospective alternatives to antibiotics based on an ecoimmunological view.

Groups	Examples*	Pros	Cons
Environmental prevention, husbandry and management techniques	Controlled therapeutic and particularly nontherapeutic antibiotic use in animal agriculture; all-in/all-out production; hygiene; drinking water quality control; **measures to decompose or remove residual antibiotics from animal food, water and waste**	Most effective and preventive measures in the long term	Requires global collaboration, huge investment, and some measures are not feasible in developing countries/areas
Pathogenic bacteria	Bacteriophages and endolysins; predatory bacteria; *metals and minerals;* **bacterial virulence inhibitors**; ***bacteriocins and antimicrobial peptides***; **pathogen-targeting aptamers; AR genes editing with CRISPR/Cas9 system; measures to eradicate persisters**	Pathogen-targeting to obtain control of epidemic infectious bacteria	All pathogen-killing measures have potential for directional selection of pathogenic resistance
*Microbial ecology*	*Bacterial growth inhibitors, or bacteriostatic metabolites; prebiotics/probiotics; fecal commensal transplantation; **bacteriocins and antimicrobial peptides; quorum sensing inhibitors; biofilm inhibitors**; **c-di-GMP and c-di-AMP***	Good for establishing inter-regulatory microbial system via community compulsion or ecological signaling	Most are based on black-box trials, there is a lack of mechanistic studies and effective measures to regulate pathogens over time
Animal immunity	Late weaning, colostrum quality and intake; egg yolk immunoglobins; antimicrobial peptides; **preventive vaccines or adjuvants**; ***innate immune signaling molecules; regulation on immunity seasonality***	Host immunity-centered on promoting animal health in general or disease-targeting, to avoid AR development	Cost and challenges for developing cross-protective vaccines and immunological measures
Animal metabolism, physiology and inter-systemic interaction	*Metals and minerals*; essential oils; amino acids; **nucleotides** (including ***c-di-GMP, c-di-AMP, and cGAMP)***; enzymes; bioactive food additives including plant, and yeast extracts; *innate immune signaling molecules;* **short-chain fatty acids; FDA-approved drugs working on G-protein coupled receptors and calcium signaling**	Synergistic promotion of both animal growth and health, less chance to develop AR	Less effective during disease epidemics or pandemics
Host-pathogen interaction	***Quorum sensing inhibitors*; Compounds that inhibit bacterial adhesion; *c-di-GMP, c-di-AMP, and cGAMP;* aptamers or other inhibitors to intervene in host-pathogen interaction**	A non-killing measure to effectively suppress on-site infections and less chance to develop AR	Requires identification of key components mediating pathogen-host interaction, more suitable for viral diseases

*Format legends: Italic, have multiple action models, such as both bactericidal and animal regulation roles of metals and minerals; Bold, most recently studied or prospective alternatives to antibiotics germane to an ecoimmunological view. Abbreviation: AR, antibiotic resistance; c-di-AMP/GMP: cyclic diadenylate/diguanylate monophosphate; CRISPR, bacterial clustered regularly interspaced short palindromic repeats (CRISPR); Cas9 CRISPR-associated protein 9 nuclease. See Section 3 and 4 for details and related references.

*Environmental prevention, husbandry, and management techniques*: Traditionally, measures related to environmental prevention, husbandry, and hygiene management for animal health are typical tools of epidemiology or disease ecology. All of these have been shown to be essential to control epidemic infections and provide effective implications for restriction of ART prevalence [29,67,68,69,70,71,72]. Because ART is a bacterial adaptation to environmental increases in artificial antibiotics, efforts to control or reduce antibiotic levels in our ecosystem are fundamental to restoring ART evolutionary dynamics to that of the pre-antibiotic era. New measures to decompose or remove residual antibiotics from animal food, water, and waste are imperative [94]. Mechanisms of AR such as enzyme degradation and detoxification imply ways to solve AR problems [94], such as bioengineered bacteria with high beta-lactamase activity to degrade lactam antibiotics in environmental samples and thereafter to trigger a mechanism for self-destruction or editing out of the beta-lactamase gene to prevent AR transfer [29,94].

*Pathogenic bacteria-targeting*: Accompanied by the declining rate of discovery of novel antibiotics in recent years, several other antibacterial or bactericidal approaches have been postulated [26,28,32,33,35,73,74]. A prominent idea is to use bacteriophages, a group of bacterial viruses that cause lysis in host bacteria in the late phase of the life cycle, to control pathogenic bacterial species. Phage therapies have been used to treat accessible topical infections and foodborne pathogens in the United States but are limited for clinic uses by regulatory hurdles because of biosafety concerns [26,32,33]. Phages have been widely studied for their advantages of targeting specific bacteria, complementing the effect of antibiotics, and less resistance by bacteria; however, host specificity of phages limits a phage therapy to treat different bacterial species or sub-species [26,32,33]. Recent studies have shown that bacterial phages actually mediated antibiotic gene transfer to preserve their host bacteria during antibiotic stress [95]. One way to tailor therapeutic uses of phages in animal production is to engineer phages’ lytic protein component of endolysins, which possess an ability to permeablize bacterial cell walls and causes lysis [26,32,33]. Phage endolysins resemble antimicrobial peptides (AMPs), which include several classes of innate immune peptides expressed in most organisms from bacteria and plants to animals [96,97,98,99,100,101]. Distinct from metabolic antibiotics, AMPs are gene-encoding protein products and primarily target cell membranes to exert broad antimicrobial activity against bacteria, fungi, tumor cells, and enveloped viruses (see detail description about AMPs in Section 4) [102,103,104]. The other pathogen-targeting approaches include predatory bacteria, such as *Bdellovibrio*-like organisms (BALOs), that use Gram-negative bacteria for energy and nutrients. Nevertheless, similar to bacteriophage therapies, wide use of BALOs in animal agriculture requires further validation to address the concerns of microbe ecology and public health [32,33]. Remarkably, most pathogenic bacteria-targeting alternatives adopt the same tenets of traditional antibiotics, which ultimately may cause directional selection of therapeutic resistance if used alone or in excess [25,27,29]. Other strategies devised from aptamer- and gene editing approaches at the molecular level have emerged only recently but show promise for impeding resisters specifically [105,106,107,108]; this will be further discussed in Section 4.

*Microbial ecology*: Bacteria regulate their pathogenicity and community formation (such as biofilms), in part, through a quorum sensing (QS) system [109,110]. QS systems consist of self-induced signaling molecules, the autoinducers, and the cognate receptors linking to down-stream regulatory proteins. Autoinducers include N-acyl homoserine lactones (AHLs) and autoinducing peptides (AIPs) secreted from Gram-negative and Gram-positive bacteria, respectively, as well as autoinducer-2 (AI-2), quinolones, esters, and fatty acids [110]. Thereby, QS inhibitors (QSIs), including molecular analogs, QS quenching enzymes, and competitive organisms, attenuate the pathogenicity and community formation of targeted bacteria ecologically to avoid ART induction [100,109]. Many QSIs show promise in bacterial control *in vitro* and are undergoing clinic trails for validation *in vivo*. Biofilms are groups of bacteria embedded in a self-produced extracellular polymeric matrix. Bacteria within biofilms obtain increased tolerance to antibiotics and disinfectants (resisters by ecological merits) [29,110]. Therefore, QSIs and other inhibitors (including those targeting bacterial protein, DNA and RNA syntheses, and secretion processes) could suppress ART development through diminishing biofilm formation [102,109]. The other widely used means to mediate homeostasis of internal microbiota include prebiotics, probiotics, or combined synbiotics, which artificially introduce benign microorganisms or commensals to modulate internal microbial ecology [87]. The recent progress in probiotic therapies includes fecal transplantation of microbiota species from healthy animals [86,87]. Collectively, alternatives in this category control pathogenic bacteria through ecological compulsion and signaling, which include multiple QSIs [86,87,100,109,110].

*Regulators of animal immune and physiological systems*: Oversimplified antibiotic therapies neglect the role of animal immunity in animal health. Potentiation of the animal immune system and interaction with other physiological systems (including microbiota) should form the core of animal health management practices [93,100]. Alternatives such as preventive vaccines and implementation of passive immune protection from colostrum or other immunoglobulin sources have succeeded, particularly in prevention of bacterial infection in young animals [26,28,33,35,100]. Antimicrobial peptides (also termed host defense peptides) and other innate immune molecules potentiate immune responses [96,99,101,104]. However, because of the high production cost of highly purified or synthetic forms, widespread use of AMP-based immunomodulators in animal production is more practical to include in regimens with synbiotics or vaccines [99,100,101,104]. Ecoimmunology emphasizes the interaction of animal immune systems with other physiological systems. Indeed, most alternatives listed for regulation of the immune system play a dual role in other physiological systems. Alternatives including metals/minerals [35,111], amino acids [112], and nucleotides [92] are essential nutrient compounds necessary for mediating cell metabolic feedback but potentially signaling immune responses as well. For example, cyclic dinucleotides (CDNs), including cyclic diadenylate monophosphate (C-di-AMP) and C-di-guanylate monophosphate (C-di-GMP), were previously accepted as important signaling molecules in bacteria and recently noted as regulators of immune response in mammalian cells [113]. Cyclic di-GMP has been implicated in multiple processes of Gram-negative bacteria, including cell motility, metabolism, differentiation, bacterial virulence, stress survival, antibiotic production, and biofilm formation; and C-di-AMP, although less studied, has been implied to play similar regulatory roles in Gram-positive bacteria. Interestingly, C-di-GMP-AMP (cGAMP) was recently found to be synthesized by mammalian cyclic cGAMP synthase (cGAS) in response to pathogenic DNA derived from intracellular retroviruses or bacteria; in turn, CDNs (including C-di-AMP/GMP and cGAMP) activate host interferon (IFN) responses through binding to intracellular adaptor proteins [113,114]. Therefore, CDNs constitute a group of universal signaling molecules in both bacteria and host cells and show promise for development as alternatives for regulation of microbial ecology, antibiotic resistance, and host immunometabolic responses.

Other alternatives, including short-chain fatty acids, enzymatic or bioactive food, yeast extracts, cytokines, and hormones, have some overlap with prebiotics and immune regulators. Their synergistic function in regulating animal physiology, metabolism, and immunity deserves mechanistic study for further validation in animal health [26,28,33,35,100,115]. Although alternatives that can actively block bacteria-host interaction are lacking, some prospects (including QSIs, CDNs, and aptamers competing for bacterial adhesion) warrant further investigation.

## 4. Prospective Alternatives and Emerging Approaches Germane to Ecoimmunological Principles

According to ecoimmunological principles, ART prevalence should integrate evolutionary, ecological, and immunological aspects rather than only targeting bacteria. This approach requires further ecoimmunological points to illustrate prospective approaches based on recent studies of the functions of current alternatives to antibiotics (Table 1).

*Phage therapies, AR gene transfer, and AR gene editing*: Bacteriophages have been identified from most, if not all, common bacterial species with ART. Using these viral phages through nurturing natural microbial ecology to control pathogenic bacteria would be a powerful approach. Nevertheless, disadvantages of phage therapies come from our incomplete understanding of the phage–bacteria parasitic relationship, including factors that determine phages’ parasitic specificity, virulence, and genetic exchange with infected bacterial hosts. All of these factors not only limit the efficacy of phage therapies, but also causes biosafety concerns [26,28,33,35,100]. For example, the use phages as an alternative to reduce AR may be hindered by the discovery that phages, in some cases, actually mediate AR gene transfer to host bacteria [97]. Recent findings about the bacterial targeted nuclease system, CRISPR/Cas9, which consists of a Cas9 nuclease and a guided-RNA (gRNA) from the bacteria clustered regularly interspaced short palindromic repeats (CRISPR), show great potential of targeted gene-editing and bioengineering manipulation [107,108]. Through this type of manipulation, some prospective phage therapies could control pathogenic bacteria and also maintain commensal homeostasis [107,108].

*Multifunctional AMPs* vs. *only antimicrobial function*: As of May 2014, the AMP database (http://aps.unmc.edu/AP/about.php) contained over 2400 entries, 98.5% of which are from natural sources and have experimentally demonstrated sequence characteristics and antimicrobial activity [101]. AMPs serve as “natural antibiotics” and have been widely studied for development into a novel generation of antibiotics [96,97,98,99,100]. Notably, most AMPs are multifunctional, exerting antimicrobial, lipid transfer, reproduction regulation, and immunomodulatory activities. Recent studies also indicate that AMPs are critical in regulating the homeostasis of animal gut microbiota [102,103]. The multifunctional properties of AMPs (such as regulation of inflammation and modulation of immune cells) often hindered their clinical application, which focused on antibiotic action [96], but recent progress in computer-based design and structural separation of antimicrobial and immunomodulatory activities will facilitate the development of AMP-based antimicrobial therapies [104]. In addition, AMP-containing biomaterials or AMP-expressed commensals have been investigated for use as prebiotics or probiotics. From an evolutionary view, AMPs from microorganisms, insects, and plants should be more diverse in antimicrobial activity but less evolved in immune regulation than their vertebrate counterparts because of their lack of an adaptive immune system [101]. This idea seems reasonable because of the discovery that microorganisms, particularly plants, encode many more of AMPs than animals; for example, plants in the Brassica family, including Arabidopsis and rapa, have several hundred AMP genes [96,97,98,99,100,101]. These AMPs exert general or pathogen-specific antimicrobial activity and could be developed into antibiotic alternatives by themselves or function as prebiotic food additives [99,100,101,102,103,104]. More recent studies of both plant and animal AMPs, in particular the defensin superfamily, have revealed that these AMPs critically regulate reproductive processes such as sperm development and motility in both plants and animals [116,117], and that expression of animal gastrointestinal tract (GIT) defensins is closely regulated by food additives with short-chain fatty acids (such as butyrate acid) [115,118]. Together with the key regulatory role of epithelia-secreted defensins in animal GIT microbiota [103], prospective AMP-based alternatives could be directed toward antimicrobial or immunomodulatory activity, reproductive regulation, and physiological modulation through action on microbiota [103,104]. Thus, multifunction of a given immune factor in an ecoimmunological context provides multiple new solutions and strategies for development of alternatives in animal production [2,3,4,5].

*Regulation of animal immune seasonality*: It has been well documented that immune function varies on a seasonal basis [10,11]. Seasonal changes in immunity range from changes in the size and gross morphology of immunologic tissues to more subtle changes in circulating antibody concentrations and specific immune cell types [10,11,119]. For example, lymphatic tissues regress in the autumn in both hibernating and non-hibernating squirrels, but regression was more complete in hibernating animals [10,11]. Cattle produce fewer antibodies in response to an antigen in the winter than in summer [120]. There is a seasonal shift in the frequency of cells expressing Th1 cytokines (IL-2 and interferon-γ) and those expressing Th2 cytokines (IL-4) in peripheral blood mononuclear cells collected from rhesus monkeys during the winter and summer [121]. Nevertheless, all animals require a positive energy balance to survive and reproduce. Like other physiological processes, immune function is energetically expensive; the cascades of dividing immune cells, the onset and maintenance of inflammation and fever, and the production of humoral immune factors all require significant energy [10,11]. Animals presumably are more susceptible to opportunistic pathogens that cause diseases and premature death during times of reduced immune function. Although animal immune seasonality seems a common reason for the severity of many infectious diseases, few studies have sought to understand mechanistically and palliate therapeutically for season-triggered immune suppression in agricultural animals [23,28,33,100]. Management practices, including better ventilation and nutrition, may lessen animal immunity trade-offs during winter; nevertheless, therapeutic alternatives intent to maintain animal immunity during winter represent another untouched category of ecoimmunological alternatives [2,3,4,5].

*Immune potentiation via physiological regulation*: Ecoimmunology underlines the immune response and trade-off resulting from inter-systemic interaction in animals, indicating that animal health status can be potentiated through physiological regulation. Key signaling pathways, including those mediated by G protein-coupled receptors (GPCRs), intracellular calcium signals, and membrane cholesterol distribution, are equally important for the regulation of both immune and physiological responses. It is well known that immune therapies such as vaccines and antibiotics have the potential for physiological regulation [23,28,33,100]. More recently, Czyz *et al.* (2014) screened a library of 640 Food and Drug Administration (FDA)-approved non-antibiotic drugs licensed for treating psychotic, diarrheal, or angina problems [122]. They found that nearly 25% of the compounds inhibited the intracellular growth of one to three tested bacteria by 80% or more with limited toxicity to host cells, and many effective compounds targeted three cell functions related to GPCR, intracellular calcium, and cholesterol signaling as listed above [122]. This study together with those to determine antibiotics’ role in immunostimulation or growth promotion will extend the profile of prospective alternatives, not by discovering new drugs but by ascribing new functions to old drugs [54,61,62,122].

*Versatile aptamers*: Receiving their name from the Latin, aptus, meaning “to fit”, aptamers consist of optimized single-stranded RNA or DNA oligonucleotides (40–200 nt long) that bind to a wide range of biological molecular and cell targets with high affinity and specificity [105,106,123]. Due to advances of nucleic acid chemistry and high-throughput screening procedures, versatile aptamers have been characterized as ideal tools in broad applications of research, diagnosis and therapeutics because of several unique properties. In brief, aptamers have: (1) high affinity and specificity: many aptamers have disassociation constant (Kd) values ranging in pM, whereas most antibodies (Abs) range from 0.1–100 nM, which enables aptamers to discriminate between molecular enantiomers and protein isoforms; (2) efficiency in production: aptamers can be selected *in vitro* against a synthetic random oligo library (generally containing >10^18^ oligos) and validated thereafter within 2–4 weeks, in contrast to antibody production, which occurs ambiguously in animals for several months; (3) stability with almost unlimited shelf-life in contrast to comparable protein reagents; and (4) no evidence of immunogenicity to avoid the side-effect of immunological complication [105,106,123]. Promising aptamer-related studies have increased after the first FDA approval of an aptamer-drug, Macugen, to treat age-related macular degeneration in 2004 [123]. With the growth in aptamer-based companies and the range of developed products, it is likely that aptamer products will be developed to target pathogenic bacteria, particularly related to ART. In our view, aptamers, based on their property of manageable affinity for targets, represent an effective weapon for intervening in the pathogen–host interaction without killing bacteria, which should result in much lower ART response than antibiotic or antimicrobial alternatives [105,106,123].

*Regimens for eradication of resisters and persisters*: Synergistic and combined strategies underlie the central idea of ecoimmunological tenets. Some effective regimens that serve as good examples of ecoimmunology have been used or proposed to eradicate resisters and persisters and alleviate ART problems [26,28,29,30,33,35,87,100]. Those for eradication of resisters include: (1) “cycling” use of available antibiotics to reduce continual selection pressure: this involves periodic replacement of front-line antibiotics of therapeutic use to prolong the effective life of front-line antibiotics and thus keep more “alternatives” in toolkits; (2) mode-of-action-guided chemical modification of antibiotic compounds to make them refractory to certain AR mechanisms; (3) combined use of inhibitors to suppress bacterial enzymes and degrade antibiotics; for example, the combination of calvulanic acid and other potent inhibitors of β-lactamase with β-lactam antibiotics have successfully bypassed β-lactamase-mediated AR; and (4) tactics of using antibiotics with different modes of action, such as β-lactam plus an aminoglycoside or tetracycline, to treat AR emerging with AIDS or cancers [25,27,29,30,53]. Unlike resisters, persisters obtain multidrug tolerance via the adaption of growth modes and metabolic statuses. Recent studies have helped to envision some treatments to eradicate persisters by sensitizing bacterial antibiotic responses. Collins’s group found that simple sugars like mannitol, fructose, or glucose could increase the proton motive force in the bacterial membrane and decrease the number of persisters by >1000-fold when combined with gentamicin, a generic aminoglycoside antibiotic [124,125]. Conversely, Kim *et al.* (2011) isolated a compound using high-throughput chemical screening, which considerably shortened the lag time for *E. coli* persisters to resume growth and facilitated bacterial killing enforced by β-lactam or fluoroquinolone antibiotics [126,127]. It is noteworthy that combinative regimens are not simply putting together treatments but require essential pharmacodynamics studies and animal validation; in some cases, combinative approaches can result in unforeseen complexity and antagonism between drugs acting through different modes.

## 5. Summary and Remarks

Ecoimmunology introduces a new discipline in studies of animal health and immunology extending immunologists’ views to the natural context in which animals live and co-evolve. Antibiotic resistance and tolerance in bacteria are natural evolutionary consequences that have been enriched by both nosocomial and agricultural use of antibiotics due to over-interpretation of pathogen killing, which overwhelms other ecological and immunological factors. An ecoimmunological approach should be considered for alternatives to antibiotics to promote the discovery of prospective approaches to relieve ART problems and improve animal health.
